# Endoscopic Evaluation of Symptomatic Patients following Bariatric Surgery: A Literature Review

**DOI:** 10.1155/2012/753472

**Published:** 2012-05-15

**Authors:** Miral Subhani, Kaleem Rizvon, Paul Mustacchia

**Affiliations:** Department of Gastroenterology, Nassau University Medical Center, 2201 Hempstead Turnpike, East Meadow, NY 11554, USA

## Abstract

Obesity is an epidemic in our society, and rates continue to rise, along with comorbid conditions associated with obesity. Unfortunately, obesity remains refractory to behavioral and drug therapy but has shown response to bariatric surgery. Not only can long-term weight loss be achieved, but a majority of patients have also shown improvement of the comorbid conditions associated with obesity. A rise in the use of surgical therapy for management of obesity presents a challenge with an increased number of patients with problems after bariatric surgery. It is important to be familiar with symptoms following bariatric surgery, such as nausea/vomiting, abdominal pain, dysphagia, and upper gastrointestinal bleeding and to utilize appropriate available tests for upper gastrointestinal tract pathology in the postoperative period.

## 1. Introduction

 According to the National Health and Nutrition Examination Survey (NHANES), a significant amount of people living in the United States are considered overweight (BMI ≥ 25–29 kg/m^2^) or obese (BMI ≥ 30 kg/m^2^) [1]. Obesity is associated with an increased risk of diabetes, hypertension, dyslipidemia cardiovascular disease, gastroesophageal reflux disease, obstructive sleep apnea, and mortality. Adult obesity rates continue to rise which is linked with an increased risk of these comorbidities [2]. There are several treatment options available for obesity such as diet and exercise, pharmacological therapy, and surgery. Most behavioral and pharmacological treatments are not helpful in maintaining long-term weight reduction [3]. Weight loss through surgery is important not only for cosmetic purposes, but several studies have shown that weight loss achieved after gastric bypass surgery has shown reduction in the comorbid conditions associated with obesity [4, 5].

 Gastric bypass surgery is currently the only effective treatment option available for achieving prolonged weight-loss [6]. Weight loss surgery has advanced over the years with jejunoileal bypass being one of the earlier attempts at surgical weight reduction. Then came about laparoscopic vertical banded gastroplasty (LVBG), followed by Roux-en-Y gastric bypass surgery (RYGB), which is now the most commonly performed bariatric surgery [7–10]. Laparoscopic sleeve gastrectomy (LSG) is now becoming more popular in that it is a single-stage operation for treatment of morbid obesity and requires less postoperative followup. Also, morbidity and long-term weight loss of LSG are comparable to that of RYGB and adjustable gastric band, adding to its appeal. 

## 2. Complications of Gastric Bypass Surgery

 Though successful in maintaining long-term weight loss and decreasing comorbid conditions associated with obesity, gastric bypass also leads to several postoperative upper gastrointestinal (UGI) complications. These symptoms can present within a few days of the surgery up to several years postoperatively. These symptoms require further evaluation through upper endoscopy, and nearly 70% of these symptomatic patients are found to have an abnormal endoscopic evaluation related to their surgery [11, 12].

An increasing number of obese and morbidly obese patients are being referred for RYGB procedures, thus increasing the number of patients requiring endoscopic evaluation for postoperative UGI symptoms. According to the American Society for Bariatric Surgery, the number of bariatric procedures performed has increased from 23,000 in 1997 to over 63,000 in 2002 [7]. It is necessary that endoscopists familiarize themselves with these postoperative UGI symptoms and endoscopic findings in the bariatric surgery patient population.

## 3. Symptoms

Symptoms experienced by postoperative bariatric surgery patients are typically vague and overlap (Table 1), thus making it difficult to presume the cause of their symptoms. Further investigation through upper endoscopy and upper gastrointestinal series is helpful to make an accurate diagnosis.

### 3.1. Abdominal Pain

Abdominal pain is amongst one of the more common problems to occur after bariatric surgery. Abdominal pain specific to gastric bypass presents a diagnostic challenge in that the differential diagnosis is diverse. The majority of these patients will require evaluation through upper endoscopy.

#### 3.1.1. Behavioral, Dietary Disorders

Overeating and rapid eating are common causes of abdominal pain early after bariatric surgery. In this patient population, satiety is distorted so the patients' pouch will distend to the point of pain before they sense fullness. Postprandial pain along with a detailed evaluation of eating habits makes the diagnosis of rapid eating more likely. Typically these symptoms do not carry on beyond six months of surgery since most patients pick on this pattern and learn to eat correctly [13]. Certain foods, such as rice, pasta, breads, and fibrous foods (e.g., meats, some vegetables, and fruits) may cause a brief impaction resulting in pain. This is more commonly seen with banded gastric bypass [14]. The appropriate treatment is to either eat these foods in smaller amounts or to avoid completely.

#### 3.1.2. Functional Disorders

 Constipation in the postoperative period is typically associated with crampy abdominal pain localized to the lower abdomen. This is caused by dehydration in the early postoperative period, and treatment is simple with increase in oral hydration and temporary laxative use. Patients with underlying functional disorders (e.g., chronic constipation and irritable bowel syndrome) can have worsening of their symptoms after gastric bypass.

#### 3.1.3. Biliary Disorders

 Patients presenting with colicky abdominal pain after extreme weight loss following bariatric surgery are highly suspicious for having cholelithiasis. Prevalence of cholelithiasis in this patient population was greater than 40% [15]; these rates have been reduced to less than 3% after the addition of ursodiol prophylaxis [15]. Some bariatric surgeons prefer removing the gallbladder prophylactically to either remove gallstones that are present or prevent gallstone formation. Whereas other surgeons prefer to perform a cholecystectomy only in the setting of gallbladder pathology. However, there are no definite guidelines on whether prophylactic cholecystectomy should be performed. Unfortunately, cholecystectomy performed during laparoscopic bypass surgery has shown an increased rate of unfavorable outcomes postoperatively. Some studies found that this procedure increases in-hospital mortality and is associated with higher rates of infections and pulmonary and gastrointestinal complications [16]. The amount of patients undergoing laparoscopic gastric bypass with an accompanying cholecystectomy has decreased over time and should be reserved for patients with symptomatic gallbladder disease. Choledocholithiasis and gallstone pancreatitis are less-common causes of abdominal pain. Endoscopic retrograde cholangiopancreatography (ERCP) is a technically difficult procedure in patients with RYGB patients due to difficult access of the ampulla. Sleeve resection eliminates this handicap by maintaining the normal anatomical route to the ampulla through the pylorus.

#### 3.1.4. Ulcer Disease

 Ulcers may occur within the gastric pouch or at the gastrojejunal anastomosis at any point after gastric bypass. These ulcers can be caused by excessive tension at the anastomotic site or due to the formation of a gastrogastric fistula, and retained pouch parietal cells. Gastrogastric fistulas typically occur in the setting of an incompletely divided pouch, and the remnant stomach's acid secretion causes ulcers. The retained pouch typically has a large amount of parietal cells along the distal lesser curvature leading to anastomotic ulcers. Decreasing the curve, creating a smaller, shorter pouch and eliminating retained pouch parietal cells may reduce the formation of ulcers. Other less common causes of ulcers include NSAID use, *Helicobacter Pylori* infection, and use of nonabsorbable sutures or staples.

#### 3.1.5. Anastomotic Leak

**Figure 1:**
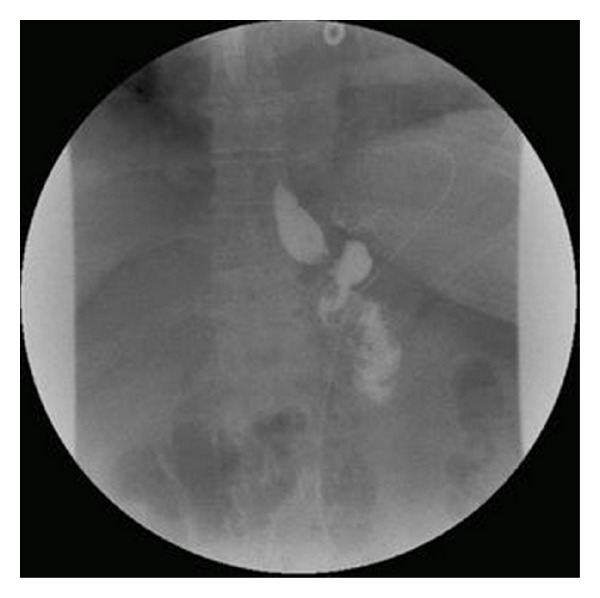
Normal postoperative contrast study.

**Figure 2:**
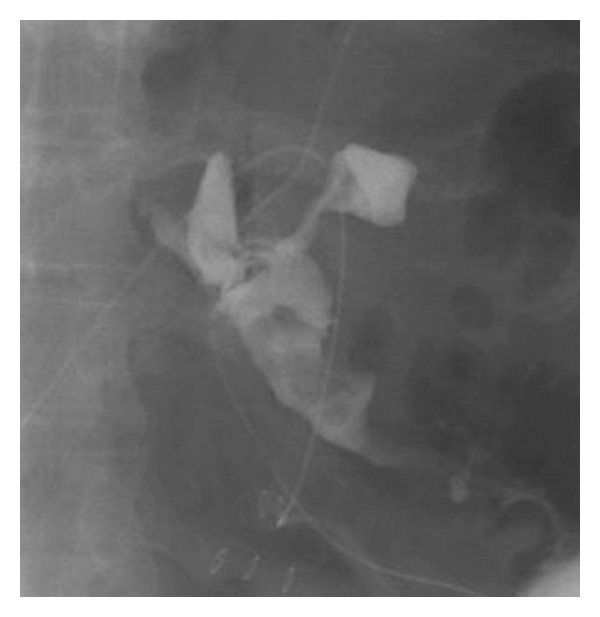
Leak with extravasation of contrast.

 Anastomotic leaks are associated with high morbidity and mortality. Anastomotic leak is one of the leading causes of death following gastric bypass surgery and sleeve gastrectomy. The incidence of anastomotic leak ranges from 0 to 5.2%, higher in those with previous bariatric operations. Most common site of these leaks after gastric bypass is the gastrojejunostomy area and proximal one-third of the stomach near the gastroesophageal junction in patients with sleeve gastrectomy [17]. Risk factors for anastomotic leaks after sleeve gastrectomy include poor suture line healing and ischemia near the staple line caused by tissue damage when using electrocautery equipment. Early diagnosis is key and cannot be based on clinical symptoms alone. Ballesta et al. reported that 49.2% of patients were asymptomatic at time of diagnosis [18]. In patients who are symptomatic, abdominal pain is the most common complaint, followed by fever. Clinical studies have shown tachycardia as being the most sensitive indicator of an anastomotic leak [19, 20]. Evaluation by UGI contrast study will show extravasation at the site of the leak (Figures 1 and 2) [21]. Early detection can be achieved through drain placement near the gastrojejunostomy or placement of a removable covered esophageal stent in the gastric sleeve across the site of anastomotic leak in patients with sleeve gastrectomy.

 Other less common causes of abdominal in postoperative bariatric surgery include internal hernias, intussusceptions, and stenosis of jejunojejunostomy.

### 3.2. Nausea

**Figure 3:**
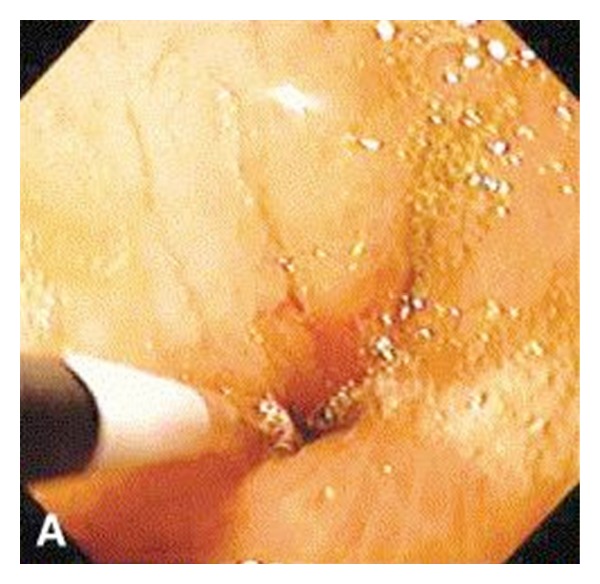
Endoscopic view showing marked stomal stenosis requiring dilatation [11].

 Nausea is a very common, vague complaint felt by many patients postoperatively and poorly correlates with endoscopic findings. Most patients who experience nausea with vomiting and epigastric discomfort have normal postoperative endoscopic findings. Patients who have marginal ulcers most often have a chief complaint of nausea and vomiting. Nausea in the setting of dysphagia is often a chief complaint of those with stomal stenosis (Figure 3). Though patients with nausea typically have a normal endoscopic workup, this symptom should not be ignored as it may indicate a more serious pathology.

### 3.3. Gastroesophageal Reflux

Bariatric surgery alters UGI anatomy which can cause symptoms of GERD postoperatively. Acid reflux occurs more often in vertical banded gastroplasty and laparoscopic adjustable gastric banding (LAGB) as compared to RYGB [22]. Vertical banded gastroplasty consists of removing the greater curvature of the stomach and creating a long tubular gastric sleeve [23]. This creates a motility disturbance in the upper GI tract. Also, there are several pathology reports showing that sling fibers of the lower esophageal sphincter are often resected during the procedure leading to a decrease in lower esophageal sphincter pressure [24, 25]. Lack of gastric compliance, severely restricted gastric capacity with an intact pylorus, and impaired gastric emptying are additional suspected predispositions for GERD-like symptoms during the postoperative period [26, 27].

### 3.4. Upper GI Bleed

The incidence of upper gastrointestinal bleed following laparoscopic gastric bypass surgery ranges from 1% to 4% [28] which may be due to penetration of tissue at the staple line, or more commonly a bleeding marginal ulcer at the site of the gastrojejunal anastomosis. There are 4 possible sites of hemorrhage at the staple lines: the gastric pouch, the gastrojejunostomy, the jejunojejunostomy, and the staple lines of the bypassed stomach. The incidence of marginal ulcers in this patient population is as high as 7% [29]. One study showed that, out of 155 patients who underwent laparoscopic RYGB, five [3.2%] developed gastrointestinal (GI) hemorrhage. Two patients presented with hematemesis, 1 patient with hypotension, 1 patient with melena, and 1 patient with bright red blood per rectum. In 2 of the 5 patients, hemorrhage occurred within 12 hours [30]. Several studies compared the incidence of GI hemorrhage in open versus laparoscopic RYGB and concluded that the frequency of GI hemorrhage is greater in laparoscopic than that of open RYGBP. One explanation is that open gastrojejunostomy anastomosis is hand sewn which decreases the likelihood of suture-line bleeding when compared to stapled anastomosis [30]. It has been found that etiology of UGI bleed can be predicted depending on the timing of onset. Early postoperative UGI hemorrhage is likely due to site at gastrojejunostomy, gastric remnants, or jejunojejunostomy staple lines [28]. Late presenting GI bleed is more commonly caused by marginal ulcer at the gastrojejunostomy site. In one study, the 16% of patients found to have staple-line dehiscence commonly complained of abdominal pain [7]. Though in this study only 1 patient with staple line dehiscence also was found to have a marginal ulcer, previous studies have shown a close association between these 2 complications [31, 32].

### 3.5. Dysphagia

Dysphagia is a troubling complication in the bariatric surgery patient. Upper endoscopy is a useful tool to assess this complaint. In one study, the presenting symptom of dysphagia correlated with an abnormal upper endoscopy in nearly two-thirds of patients while abdominal pain was associated with normal endoscopy in nearly half of patients [33]. Another study determined that patients who presented with UGI bleed and dysphagia had the most remarkable endoscopic abnormalities [34].

#### 3.5.1. Stenosis

 Often, anastomotic stenosis occurs within 3 months of bariatric surgery. The most common presenting symptom is dysphagia. However, when coupled with ulcer disease or anastomotic leak, these patients may also present with significant abdominal pain. Incidence of anastomotic stenosis ranges from 5% to 10% [35]. The cause of strictures is unknown in most patients; however, there have been some factors identified that increase the risk of these strictures. One study showed that using a circular stapler increased the rate of stricture formation as compared with hand-sewn or linear stapling anastomosis from 3% to 31% [36]. Another study suggested that most refractory anastomotic strictures have been secondary to excessive gastric acid exposure. The anastomosis is exposed to an inappropriately large-volume proximal gastric pouch containing acid secretory mucosa resulting in ongoing inflammation, ulcer, and stricture formation.

#### 3.5.2. Esophageal Dilatation/Esophageal Motor Dysfunction

Esophageal dysmotility is complication seen often in laparoscopic gastric banding. In a recent study, 167 patients were followed from 1998 to 2009, esophageal dysmotility disorders were found in 108 patients [68.8%], and esophageal dilatation occurred in 40 patients [25.5%]. In 29 patients, upper endoscopy was performed because of heartburn or dysphagia with a normal endoscopy in 18 patients and 9 with evidence of GERD. The high incidence of dysphagia caused by esophageal dilatation and motility disturbance is a troublesome complication of LAGB [37]. 

## 4. Method of Evaluation

Upper Endoscopy and Upper GI series are the initial tests of choice. In a study of 1,076 patients who underwent RYGB, 76 developed UGI symptoms postoperatively [33]. Out of those 76 patients, 36 patients were initially evaluated by UGI series (UGIS) for their symptoms with 13 abnormalities: 12 with a stricture and 1 with gastrogastric fistula. The UGIS recognized 12 of the 18 patients [67%] with anastomotic strictures and did not identify any of the three patients with marginal ulcers. The overall diagnostic yield of the UGIS in this study was found to be 64.9% [33]. Compared to upper endoscopy, the sensitivity and specificity of the UGIS for detecting abnormalities related to bariatric surgery were 64% and 85%, respectively.

 While upper endoscopy identified marginal ulcers and esophagitis more consistently than UGI series, it did not identify anastomotic narrowing. With UGI series, the flow of the contrast across the anastomotic site provides valuable information on the size of the stoma. This information may assist in the diagnosis of less significant strictures which may be missed by the passage of small caliber endoscopes during upper endoscopy [7].

## 5. Endoscopic Findings After Bariatric Surgery

Though there are no set guidelines, indications for endoscopic evaluation in postoperative bariatric patients consist of evaluation of presenting symptoms. The majority of patients has an unremarkable examination. Abnormal findings are defined as erosive esophagitis, esophageal ulcer, gastric ulcer, marginal ulcer at the gastrojejunal (G-J) anastomotic site, anastomotic stricture, excess suture material causing obstruction at the G-J anastomosis, gastrogastric fistula, jejunal ulcer, and food impaction (Table 2).

### 5.1. Normal Postoperative Findings

Several studies have shown that the majority of patients who underwent endoscopic evaluation for UGI symptoms postoperatively were found to have normal postoperative endoscopic findings, which is consistent with previous reports [7, 38]. These patients present mostly with nausea/vomiting, epigastric discomfort, and gastroesophageal reflux [34]. It is likely that these symptoms were attributed to the small gastric pouch not being able to accommodate a large amount of food. Patients' eating habits included consuming large food boluses in the setting of inadequate chewing [8, 39]. It is important to educate patients regarding etiology of their symptoms and how to manage them appropriately by modifying eating habits to decrease recurrence of these symptoms long-term. Yang et al. evaluated 160 symptomatic patients with upper endoscopy and found a normal anatomy as being the most coming findings [*n* = 57; 46 in LVBG, 11 in LRYGB], which is consistent with previous reports [7, 38].

### 5.2. Ulcers

**Figure 4:**
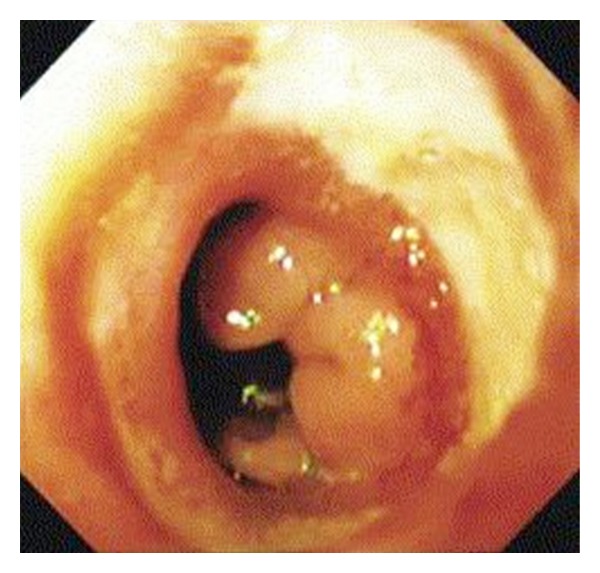
Endoscopic view showing marginal ulcer [11].

The most common post-RYGB complications reported in literature are marginal ulcers [27–52%] (Figure 4), anastomotic strictures [4–27%], and staple-line disruption [4–16%] [7, 31, 40–45]. Development of these complications is attributed to variations in surgical techniques [31, 46].

One study that evaluated 72 symptomatic patients found that of those who presented with marginal ulcers, the majority complained of nausea/vomiting as their chief complaint [*n* = 7, 58.3%] [33]. Another center followed 104 symptomatic patients in which 26 situations presented with UGI bleeding [34]; 23 of those were attributed to marginal ulcers and 3 to esophageal ulcers. Most of the patients who developed marginal ulcers presented with UGI bleed [60%] and epigastric discomfort [33.5%]. Ulcers were also the most common finding in the symptomatic patients of another community hospital-based study of 200 patients [12]. Another important finding is the absence of the association between *Helicobacter pylori* and ulcers seen in the bariatric surgical patient. In this study all patients undergoing endoscopy tested negative for *Helicobacter pylori*.

### 5.3. Anastomotic Strictures

Though the cause of anastomotic strictures remains unclear, there are several factors that contribute to the development of this complication including tension on the anastomosis, ischemia, type of stapler, and healing capacity of individual. These strictures tend to develop early likely due to local inflammation at the site of the anastomosis and decreased blood supply leading to fibrosis [33].

### 5.4. Food Impaction

Bariatric surgery alters gastric motility resulting in food impaction. Though stomal stenosis can result in impaction, this finding was found in just one of the 21 patients who presented with impaction in a large study of 1090 patients. Most common foods impacted were meat bolus [14/21, 66.7%] and plum seeds [4/21, 19%]. Educating patients regarding appropriate diet, including avoiding certain foods most likely to cause impaction and obstruction, is essential for prevention of this complication [34].

Data is compared between patients with normal and abnormal endoscopic findings in Table 3. Patients that present with symptoms of dysphagia and those with UGI bleed tend to have abnormal endoscopic findings.

## 6. Timing of Onset of Symptoms

 One study showed that the average time interval from surgery to initial endoscopy was 185.5 days. This same study showed that the average time interval from surgery to presentation of UGI symptoms specifically in patients with abnormal findings at endoscopy was a mean of 110.7 days compared to a mean of 347.5 days in patients with normal postoperative endoscopic findings. More patients were found to have endoscopic abnormalities when the exam was performed after 90 days of the bariatric surgery. Of those patients undergoing endoscopy within the first 3 months after bariatric surgery, only 14% had normal findings compared to 47.4% of patients after 3 months. Marginal ulcers were seen in 4.7% of patients who had upper endoscopy less then 3 months after surgery compared to 26.3% who had upper endoscopy after 3 months of the surgery. However, anastomotic strictures were more common in the symptomatic patient in the first 3 months [33].

## 7. Conclusion

 Bariatric surgery is the most effective treatment for morbid obesity and is proven to be successful in maintaining long-term weight loss. Laparoscopic RYGB is the most commonly performed bariatric surgery, with laparoscopic sleeve gastrectomy gaining popularity. An increase in weight reduction surgery has revealed complications related to bariatric surgery and certain symptoms that correlate to these complications.

 UGI symptoms following bariatric surgery, including nausea and vomiting, UGI bleed, dysphagia, and abdominal pain require investigation. UGI series is the preferred initial test; however, endoscopy is the most accurate diagnostic method. Endoscopy is proven to be safe postoperatively and an effective method for treatment of pathology causing UGI symptoms after bariatric surgery. Abdominal pain is a common complaint with a wide differential diagnosis and endoscopic evaluation as these patients may reveal ulcer disease, stomal stenosis, or anastomotic leaks. Patients with normal upper endoscopy and abdominal pain develop these symptoms due to poor eating habits and need to be educated on proper diet. UGI bleed is usually indicative of an ulcer, typically if it occurs after three months following bariatric surgery; if UGI bleed occurs within three months of the procedure, hemorrhage at the staple line is usually the cause. Dysphagia within three months of bariatric surgery is a common chief complaint of those found to have anastomotic stenosis. Most patients who undergo endoscopic evaluation of UGI symptoms after gastric bypass are found to have normal anatomy. Common abnormal findings include marginal ulcer, anastomotic stenosis, and staple line disruption. Bariatric surgery alters gastric motility resulting in food impaction, and educating patients regarding appropriate diet can decrease the incidence of this complication.

There are no set guidelines on when to endoscopically evaluate patients after bariatric surgery. Thus recognizing symptoms postoperatively and being familiar with the spectrum of endoscopic abnormalities related to bariatric surgery will aid in early diagnosis and treatment.

## Figures and Tables

**Table 1:** UGI symptoms following bariatric surgery.

Symptoms	Yang et al.*	Huang et al.**	Lee et al.***
Nausea/vomiting	47 [29.4%] LVBG = 38; LRYGB = 9	26 [53%]	37 [48.7%]
Epigastric discomfort	44 [27.5%] LVBG = 32; LRYGB = 12	—	—
UGI bleed	26 [16.3%] LVBG = 7; LRYGB = 19	6 [12%]	1 [1.3%]
Abdominal pain	—	26 [53%]	19 [25%]
Heartburn/acid regurg	26 [16.3%] LVBG = 22; LRYGB = 4	—	—
Dysphagia	10 [6.3%] LVBG = 10; LRYGB = 0	8 [16%]	41 [53.9%]
Anemia with dizziness	7 [4.4%] LVBG = 2; LRYGB = 5	—	—

*A total of 104 patients underwent endoscopic examinations, 76 patients had undergone LVBG, and 28 had undergone LRYGB.

**This study describes patients who underwent LRYGB.

***This study describes a total of 76 patients who underwent RYGB: 66 underwent LRYGB, 6 underwent open RYGB, and 4 underwent robotic RYGB.

**Table 2:** Endoscopic findings for evaluation of UGI symptoms after bariatric surgery.

Findings	Yang et al.	Huang et al.	Lee et al.*
Normal	57 [35.6%] LVBG = 46; LRYGB = 11	21 [43%]	24 [31.6%]
Marginal ulcer	39 [24.4%] LVBG = 8; LRYGB = 31	12 [27%]	12 [15.8%]
Gastric ulcer	7 [4.4%] LVBG = 7; LRYGB = 0	—	—
Duodenal ulcer	1 [0.6%] LVBG = 1; LRYGB = 0	—	—
Esophagitis/esophageal ulcer	21 [13.1%] LVBG = 20; LRYGB = 1	2 [4%]	—
Food impaction	21 [13.1%] LVBG = 18; LRYGB = 3	—	—
Stenosis or stricture	14 [8.8%] LVBG = 11; LRYGB = 3	9 [19%]	40 [52.6%]
Staple line dehiscence	—	8 [16%]	—

*Normal endoscopic findings: laparoscopic: 20, open: 3, robotic: 1; abnormal endoscopic findings: laparoscopic: 46, open: 3, robotic: 3.

**Table 3:** Comparison of patients with normal versus abnormal findings.

	Normal endoscopy			Abnormal endoscopy		
Symptoms	Lee et al.	Huang et al.	Yang et al.	Lee et al.	Huang et al.	Yang et al.
Nausea/vomiting	13	8	18	24	9	29
Epigastric discomfort	—	—	23	—	—	21
UGI bleed	0	1	0	1	5	26
Heartburn/acid regurg	—	—	12	—	—	14
Dysphagia	6	3	1	35	5	9
Abdominal pain	11	15	—	8	11	—
